# Sphingosine-1-phosphate receptor 1 activation in the central nervous system drives cisplatin-induced cognitive impairment

**DOI:** 10.1172/JCI157738

**Published:** 2022-09-01

**Authors:** Silvia Squillace, Michael L. Niehoff, Timothy M. Doyle, Michael Green, Emanuela Esposito, Salvatore Cuzzocrea, Christopher K. Arnatt, Sarah Spiegel, Susan A. Farr, Daniela Salvemini

**Affiliations:** 1 Department of Pharmacology and Physiology, and; 2 The Henry and Amelia Nasrallah Center for Neuroscience, Saint Louis University School of Medicine, St. Louis, Missouri, USA.; 3 Department of Internal Medicine-Geriatrics, Saint Louis School of Medicine, St. Louis, Missouri, USA.; 4 Department of Chemistry, Saint Louis University, St. Louis, Missouri, USA.; 5 Department of Clinical and Experimental Medicine and Pharmacology, University of Messina, Messina, Italy.; 6 Department of Biochemistry and Molecular Biology, Virginia Commonwealth University School of Medicine, and the Massey Cancer Center, Richmond, Virginia, USA.

**Keywords:** Neuroscience, Oncology, Cancer, Memory, Neurodegeneration

## Abstract

Cancer-related cognitive impairment (CRCI) is a major neurotoxicity affecting more than 50% of cancer survivors. The underpinning mechanisms are mostly unknown, and there are no FDA-approved interventions. Sphingolipidomic analysis of mouse prefrontal cortex and hippocampus, key sites of cognitive function, revealed that cisplatin increased levels of the potent signaling molecule sphingosine-1-phosphate (S1P) and led to cognitive impairment. At the biochemical level, S1P induced mitochondrial dysfunction, activation of NOD-, LRR-, and pyrin domain–containing protein 3 inflammasomes, and increased IL-1β formation. These events were attenuated by systemic administration of the functional S1P receptor 1 (S1PR1) antagonist FTY720, which also attenuated cognitive impairment without adversely affecting locomotor activity. Similar attenuation was observed with ozanimod, another FDA-approved functional S1PR1 antagonist. Mice with astrocyte-specific deletion of *S1pr1* lost their ability to respond to FTY720, implicating involvement of astrocytic S1PR1. Remarkably, our pharmacological and genetic approaches, coupled with computational modeling studies, revealed that cisplatin increased S1P production by activating TLR4. Collectively, our results identify the molecular mechanisms engaged by the S1P/S1PR1 axis in CRCI and establish S1PR1 antagonism as an approach to target CRCI with therapeutics that have fast-track clinical application.

## Graphical abstract

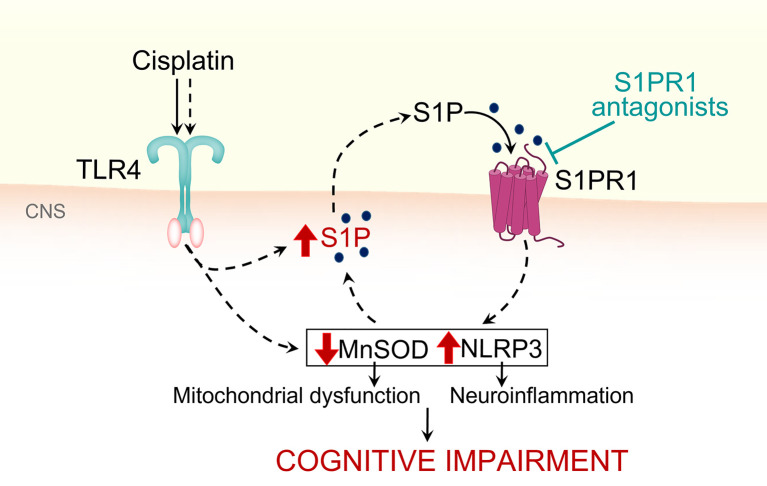

## Introduction

With major advances in cancer treatment, the National Cancer Institute expects cancer survivorship to reach 21.7 million by 2029 (1). However, cancer treatment is often associated with severe long-lasting neurotoxic side effects. Cancer-related cognitive impairment (CRCI) is a major neurotoxicity of the platinum-based drug cisplatin, widely used in treating numerous cancers (2). CRCI profoundly affects patient quality of life and is characterized by subtle to moderate cognitive deficits, including impaired processing speed, memory, executive functioning, and attention (3, 4). Reported cognitive deficits affect up to 75% of patients treated with chemotherapy for cancers outside the nervous system (4). There are no FDA-approved drugs to mitigate these deficits. Our current understanding of the mechanisms underlying CRCI and their impact on cognition is limited, due to the multifactorial origins of CRCI (5). A better understanding of these mechanisms is essential for developing therapeutic approaches and improving survivors’ quality of life. Here, we present evidence that in the central nervous system (CNS), cisplatin increases levels of the potent signaling molecule sphingosine-1-phosphate (S1P) that contributes to CRCI development through activation of S1P receptor subtype 1 (S1PR1). Mechanistically, cisplatin-induced S1P formation is mediated by Toll-like receptor 4 (TLR4). Our findings bridge gaps in our understanding of the molecular mechanisms underlying CRCI and identify a target for therapeutic intervention with functional S1PR1 antagonists. Importantly, 2 functional S1PR1 antagonists are already FDA approved for multiple sclerosis treatment: FTY720 (Gilenya, Novartis) and ozanimod (Zeposia, Celgene). Noteworthily, several preclinical studies suggest that FTY720 does not negatively interfere with the therapeutic activity of chemotherapeutics, including cisplatin, and also possesses anticancer activity by blocking tumor growth and metastasis (6–8). Moreover, we previously demonstrated in various human cancer cells that FTY720 does not alter the cytotoxic efficacy of platinum-based drugs, taxanes, and proteasome inhibitors (9). Building on a compelling preclinical platform, future clinical trials are needed to assess the anticancer effects of S1PR1 antagonists given alone or in combination with chemotherapy. Repurposing these drugs to prevent CRCI would be a ground-breaking shift toward enhancing patient quality of life in cancer treatment.

## Results and Discussion

**Figure 1:**
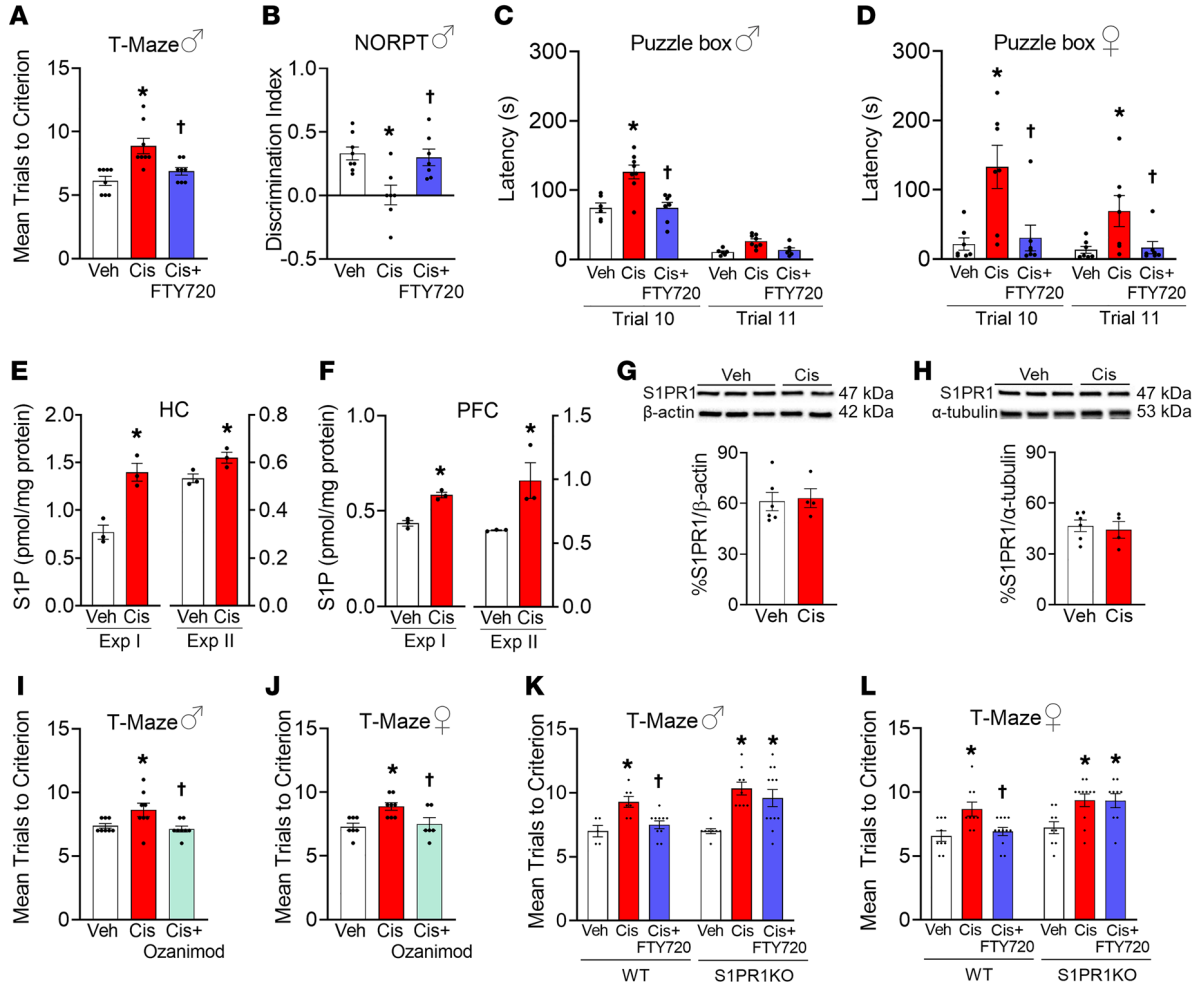
Cisplatin increased S1P in the CNS, leading to cognitive impairment that was attenuated by S1PR1 functional antagonists. (**A**–**D**) Cisplatin-treated mice (Cis, *n* = 8) showed learning, memory, and problem-solving impairments in the T-maze (**A**), NOPRT (**B**), and puzzle box test (**C** and **D**). FTY720 administered in conjunction with cisplatin attenuated cognitive deficits in both male (*n* = 8/group) and female mice (*n* = 7/group) (**A**–**D**). (**E** and **F**) Sphingolipidomics performed in WT CRCI male mice in 2 separate experiments revealed that cisplatin increased S1P in the hippocampus (HC) (*n* = 6/group) (**E**) and in the PFC (*n* = 6/group) (**F**). (**G** and **H**) S1PR1 was found expressed in the hippocampus (**G**) and PFC (**H**), placing the receptor in proximity to its ligand (*n* = 6/group). Significance tested with 2-tailed, unpaired Student’s *t* test. (**I**–**L**) The beneficial effects of FTY720 were extended to another functional S1PR1 antagonist, ozanimod, which attenuated cognitive impairment in the T-maze in both male (*n* = 8/group) (**I**) and female mice (*n* = 7/group) (**J**). The beneficial effects of FTY720 were lost in both male (*n* = 6–12/group) (**K**) and female (*n* = 7–12/group) (**L**) mice with astrocyte-specific deletion of *S1pr1* (*n* = 7–8/group). Data are presented as mean ± SEM. *P < 0.05 vs. Veh; ^†^*P* < 0.05 vs. Cis; by 2-tailed, 1-way ANOVA with Dunnett’s test (**A**–**D**), 2-tailed, unpaired Student’s *t* test (**E** and **F**), or 2-tailed, 2-way ANOVA with Bonferroni’s test (**I**–**L**). ♂, males; ♀, females.

A cisplatin treatment protocol was utilized to induce CRCI in tumor-free male and female mice (10). Consistent with a previous report (10), 2 weeks after the last dose of cisplatin, a battery of behavioral tests examining multiple aspects of cognitive function (T-maze and novel object-place recognition test [NOPRT], long-term hippocampal memory; puzzle box test, executive function) revealed that mice developed cognitive deficits (Figure 1, A–D). Mice receiving cisplatin took more trials to reach criterion in the T-maze (Figure 1A), were less likely to recognize a novel object in the NOPRT (Figure 1B), and showed memory and executive function impairments in the puzzle box test during difficult challenge (Trial 10, Figure 1C). These results were not due to reductions in overall activity or motivation to escape a noxious stimulus (Supplemental Figure 1; supplemental material available online with this article; https://doi.org/10.1172/JCI157738DS1). We previously showed that 2 chemotherapeutic agents, paclitaxel and bortezomib, cause dysregulation of de novo sphingolipid metabolism in the spinal cord that led to development of chemotherapy-induced neuropathic pain, another major cancer-treatment neurotoxicity (9, 11). These earlier findings prompted us to examine the effects of cisplatin on sphingolipid metabolites in the CNS. Two separate experiments using liquid chromatography–electrospray ionization–tandem mass spectrometry (LC-ESI-MS/MS) analysis of multiple sphingolipid species in the prefrontal cortex (PFC) and hippocampus, key centers of cognition, revealed a significant increase only in the bioactive signaling molecule S1P (Figure 1, E and F), with reductions of its precursor sphingosine (Supplemental Figure 2). There were no significant changes in the levels of ceramide and its de novo biosynthetic pathway precursor, dihydrosphingosine, nor in sphingomyelin or glycosylceramides (Supplemental Figures 2 and 3). These data suggest that cisplatin treatment does not stimulate de novo sphingolipid biosynthesis, but rather activates sphingosine kinase, the enzyme that forms S1P.

S1P is the ligand for the G protein–coupled receptor S1PR1 that we found expressed in the PFC and hippocampus, placing the receptor in proximity to its ligand (Figure 1, G and H). Cotreatment of mice with cisplatin and the functional S1PR1 antagonist FTY720 significantly attenuated CRCI (Figure 1, A–D), with no adverse effect on anxiety-like behavior and locomotor activity (Supplemental Figure 1). Similar cognitive improvements were also observed with another functional S1PR1 antagonist, ozanimod (Figure 1I), which is approved for the treatment of multiple sclerosis and has improved selectivity and a more desirable clinical safety profile than FTY720 (12). The beneficial effects observed with FTY720 and ozanimod were confirmed in female mice (Figure 1, D and J, and Supplemental Figure 1). Cisplatin, FTY720, and ozanimod did not have long-term effects on estrous cycling in female mice.

In the brain, S1PR1 is highly expressed in glia relative to neurons (13) and glial cells have been implicated in CRCI development (14). S1PR1 activation on glia facilitates release of inflammatory and neuroexcitatory substances, whereas activation on neurons increases neuronal excitability (15, 16). In the CNS, astrocytes express much higher S1PR1 levels than microglia (13), marking these cells as a prime target for S1P via S1PR1. We used conditional knockout mice in which the entire *S1pr1* open reading frame was deleted in astrocytes (Supplemental Figure 6) (17) to examine whether astrocyte-specific S1PR1 had a role in the pharmacological effects of S1PR1 antagonists. We previously confirmed that *S1pr1* deletion is restricted to the CNS (11). When compared with control littermates, mice with astrocyte-specific deletion of *S1pr1* developed CRCI to the same extent as wild-type (WT) mice, but completely lost their responsiveness to the beneficial effects of FTY720 (Figure 1, K and L). These data strongly suggest that blocking S1PR1 signaling in astrocytes is necessary for the pharmacological effects of FTY720, identifying astrocytes as a primary cellular target for S1PR1 antagonism.

**Figure 2:**
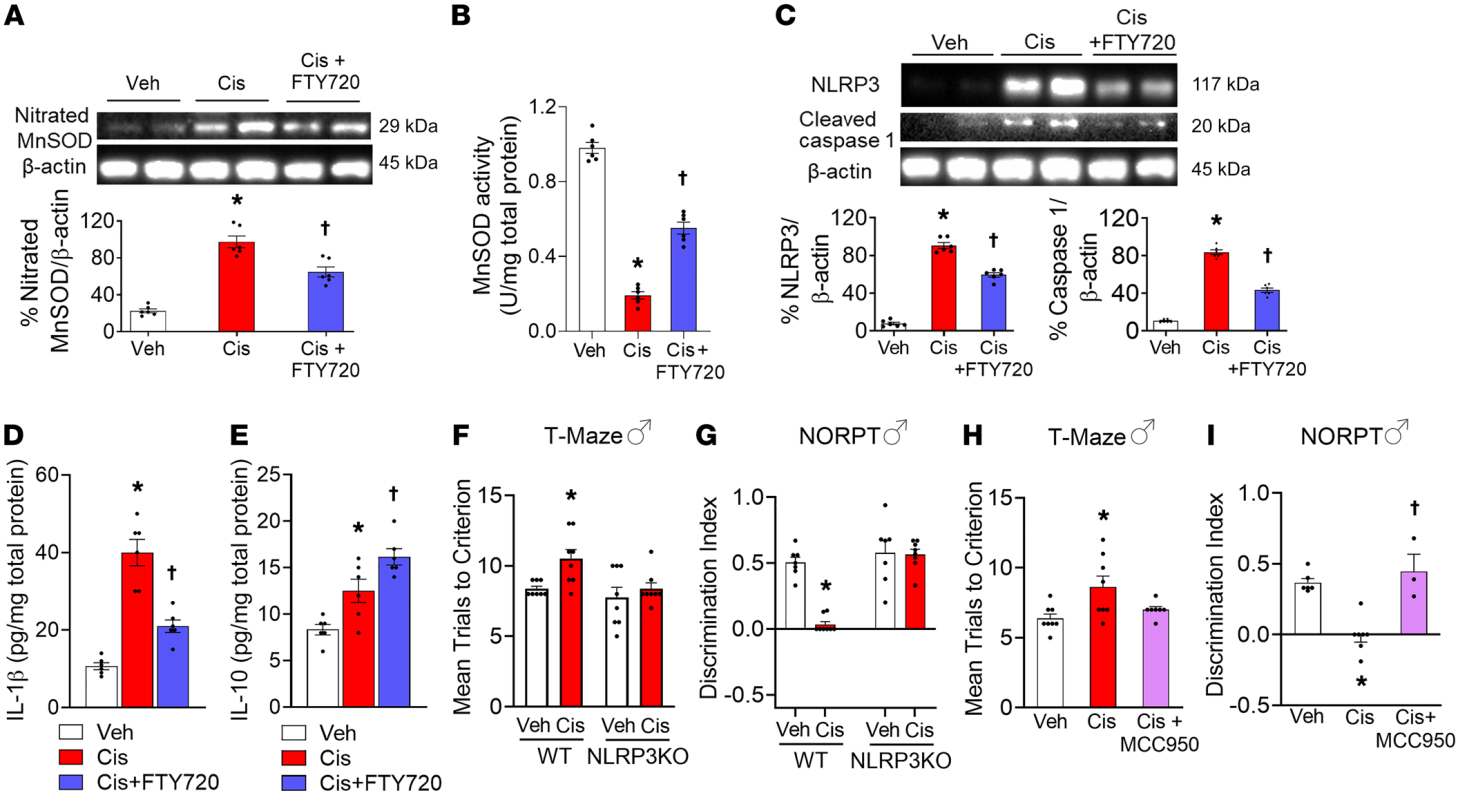
FTY720 attenuated CRCI by dampening MnSOD nitration/inactivation and NLRP3-mediated neuroinflammation, while increasing neuroprotective IL-10. Cisplatin induced an increase in MnSOD nitration (**A**) and deactivation (**B**) in the mouse hippocampus, which were attenuated by coadministration with FTY720. FTY720 also attenuated the increased expression of NLRP3 (**C**), cleaved caspase 1 (**C**), and IL-1β (**D**) in the mouse hippocampus following cisplatin. FTY720 administration increased neuroprotective IL-10 (**E**) in the hippocampus (*n* = 6/group). (**F**–**I**) Cisplatin-induced NLRP3 activation is functionally linked to CRCI, as global NLRP3–knockout (*Nlrp3*^–/–^, **F** and **G**, *n* = 7–8/group) mice or mice receiving i.c.v. infusion of the NLRP3 inhibitor MCC950 (**H** and **I**, *n* = 7/group) do not develop CRCI. Data are presented as mean ± SEM. *P < 0.05 vs. Veh; ^†^*P* < 0.05 vs. Cis; by 2-tailed, 1-way ANOVA with Dunnett’s test (**A**–**D**, **H**, and **I**) or 2-tailed, 2-way ANOVA with Bonferroni’s test (**F** and **G**).

The molecular mechanisms whereby S1P contributes to CRCI are unknown. We previously reported that direct S1PR1 activation in the CNS with highly selective S1PR1 agonists forms peroxynitrite (18), a powerful nitrating agent, and activates the NOD-, LRR-, and pyrin domain–containing protein 3 (NLRP3) inflammasome (19). Peroxynitrite nitrates mitochondrial manganese superoxide dismutase (MnSOD) at Tyr-34 via an Mn-catalyzed process that inactivates the enzyme by more than 80% and results in mitochondrial dysfunction (20, 21). In contrast, NLRP3 is critical for formation of interleukin 1β (IL-1β) and IL-18, inflammatory cytokines with known roles in cognitive impairment (22, 23). As mitochondrial dysfunction and neuroinflammation in the CNS are 2 proposed mechanisms thought to drive CRCI (14, 24), we tested potential links to S1PR1. Cisplatin led to nitration and inactivation of MnSOD (Figure 2, A and B), and NLRP3 activation (increased NLRP3 production and maturation of caspase-1 and IL-1β; Figure 2, C and D). Cisplatin-induced NLRP3 activation was functionally linked to CRCI, as global NLRP3–knockout (*Nlrp3*^–/–^) mice or mice receiving intracerebroventricular (i.c.v.) infusion of the NLRP3 inhibitor MCC950 (25) did not develop CRCI (Figure 2, F–I, and Supplemental Figure 5). We believe this is the first study documenting the roles of the NLRP3 inflammasome in cisplatin-induced cognitive impairment. These data, together with previous findings with doxorubicin (23), suggest NLRP3-driven inflammatory pathways are strongly implicated in cognitive changes following chemotherapy. Coadministration of cisplatin with FTY720 attenuates MnSOD nitration and inactivation and NLRP3 activation (Figure 2, A–D). Interestingly, FTY720 increases the levels of IL-10, a potent antiinflammatory and neuroprotective cytokine (Figure 2E). These findings are noteworthy, since genetic ablation of IL-10 is associated with neurodegeneration and cognitive decline (26), whereas exogenous IL-10 administration or pharmacological strategies that increase hippocampal IL-10 have been linked to improved cognitive function in different diseases (27–29).

**Figure 3:**
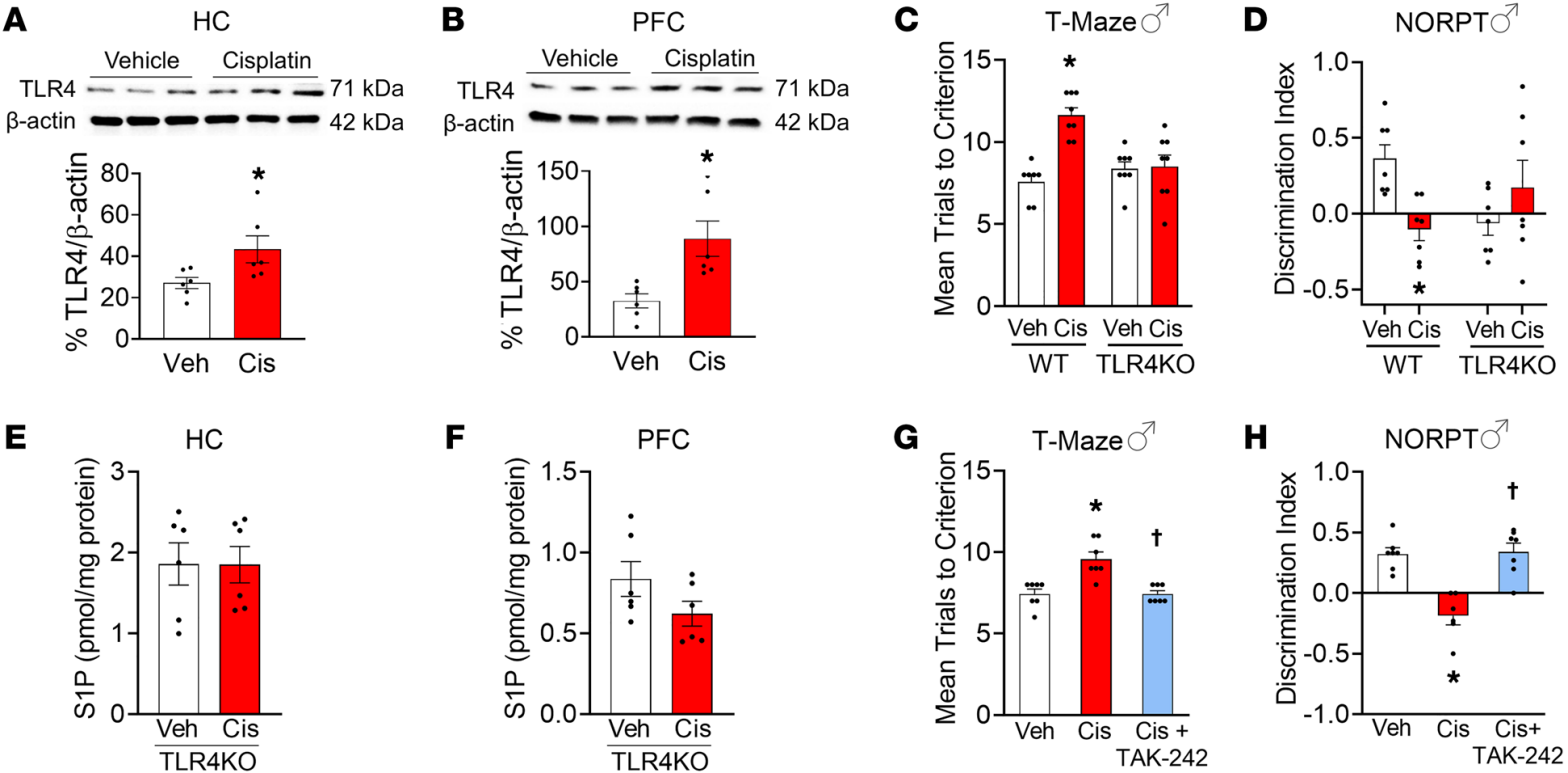
TLR4 activation in the CNS was required for cisplatin-induced S1P alterations and cognitive impairment. (**A** and **B**) In WT mice, cisplatin increased TLR4 expression in both the hippocampus (HC) (**A**) and PFC (**B**) (*n* = 6/group). (**C** and **D**) WT, but not *Tlr4^–/–^* mice, developed memory and learning deficits in the T-maze (**C**) and NOPRT (**D**) following cisplatin. (**E** and **F**) Cisplatin did not increase S1P levels in the hippocampus (**E**) and PFC (**F**) from *Tlr4^–/–^* mice, as determined by LC-ESI-MS/MS (*n* = 6/group). (**G** and **H**) WT mice receiving i.c.v. infusion of the TLR4 antagonist TAK-242 during cisplatin treatment did not develop cognitive deficit in the T-maze (**G**) and NOPRT (**H**), confirming the relevance of TLR4 activation in the CNS for the development of cisplatin-induced cognitive impairment (*n* = 7–8/group). Data are presented as mean ± SEM. *P < 0.05 vs. Veh; ^†^*P* < 0.05 vs. Cis; by 2-tailed, unpaired Student’s *t* test (**A** and **B**), 2-tailed, 2-way ANOVA with Bonferroni’s test (**C**–**F**), or 2-tailed, 1-way ANOVA with Dunnett’s test (**G** and **H**).

The molecular mechanisms whereby cisplatin triggers S1P formation are unknown. Sphingosine kinase, the enzyme involved in S1P metabolism, is activated in response to TLR4 activation (30), a membrane-bound pattern recognition receptor. TLR4 activation triggers nitroxidative stress and neuroinflammation (31, 32) and is implicated in learning and memory impairment in different pathological states (33, 34). It is now well documented that TLR4 can be activated by several different ligands, including group 9/10 transition metals, nickel, cobalt, and cisplatin (35, 36). This activation of TLR4 is independent of the myeloid differentiation factor 2 (MD-2) coreceptor and activates nuclear factor kappa-light-chain-enhancer of activated B cells (NF-κB), increases formation of IL-1β and IL-18, and leads to oxidative stress and cell death (35). The molecular mechanisms whereby cisplatin activates TLR4 are unknown. Cisplatin binds histidine residues (37) and H456 and H458 are located on the protein-protein interface of the homodimer, based on the crystal structure of the activated homodimer of the human TLR4–MD-2 complex (PDB: 3FXI) (38). Several studies showed that, along with H431, these residues form a cluster of 6 histidines in the TLR4 homodimer that may direct dimerization via binding to group 9/10 transition metals (36, 39). Our modeling of the H456/H458 cisplatin binding site showed that the histidine residues are optimally spaced within the homodimer interface to bind 2 cisplatin molecules to direct TLR4 activation via homodimerization (Supplemental Figure 4). Therefore, our results suggest that one potential mechanism whereby cisplatin activates TLR4 is by forming critical interactions with histidine residues (H456/H458) on the ectodomain of TLR4 that facilitate dimerization (Supplemental Figure 4). These results are consistent with observations using nickel and cobalt (36, 39). In addition to direct TLR4 activation, cisplatin can increase TLR4 signaling by increasing TLR4 expression (40, 41). In mice that developed CRCI, we found a significant increase in TLR4 expression in the PFC and hippocampus (Figure 3, A and B). Importantly, cisplatin lost its ability to induce CRCI in global TLR4–knockout (*Tlr4^–/–^*) mice (Figure 3, C and D). Moreover, in contrast to WT mice (Figure 1, E and F), cisplatin did not increase S1P levels in the PFC and hippocampus from *Tlr4^–/–^* mice, as determined by sphingolipidomic profiling (Figure 3, E and F, and Supplemental Figure 3). To further test whether TLR4 activation in the CNS directly contributes to CRCI, mice received i.c.v. infusions of the TLR4 inhibitor TAK-242 (42) during cisplatin treatment and then were tested for CRCI. These mice did not develop CRCI (Figure 3, G and H, and Supplemental Figure 5). We believe this is the first study implicating TLR4 activation as a causal mechanism for CRCI.

Our studies provide evidence that TLR4 activation in the brain is the linchpin in cisplatin-induced S1P formation, S1P-mediated S1PR1 activation, and cognitive deficits. Although our studies support key roles for S1P/S1PR1 in mitochondrial dysfunction and inflammasome activation, we cannot exclude contributions of S1P signaling in other mechanisms implicated in CRCI such as altered calcium homeostasis (43). Our findings establish S1PR1 as a therapeutic target and could expedite proof-of-concept clinical studies with FTY720 and/or ozanimod as adjunct to chemotherapy.

## Methods

Detailed experimental methods are included with the Supplemental Material.

### Study approval.

Procedures for the maintenance and use of animals were in accordance with the NIH *Guide for the Care and Use of Laboratory Animals* (National Academies Press, 2011) and approved by the Saint Louis University Institutional Animal Care and Use Committee and by the University of Messina Review Board for the care of animals, in compliance with Italian regulations on protection of animals (no. 368/2019-PR- released on May 4, 2019).

## Author contributions

DS conceived and designed the studies. S Squillace, MLN, TMD, EE, SC, and SAF performed the experiments and analysis. S Spiegel performed the sphingolipidomic analysis. MG and CKA performed the computational modeling studies. DS and S Squillace prepared the manuscript with input from all authors.

## Supplementary Material

Supplemental data

## Version 1 09/01/2022

Electronic publication
