# Supplementary material for: Sphingosine-1-phosphate receptor 1 activation in the central nervous system drives cisplatin-induced cognitive impairment
Source: J Clin Invest. 2022 Sep 1;132(17):e157738. doi: 10.1172/JCI157738 (PMC9433103; doi:10.1172/JCI157738)
Supplement: Supplemental data [file jci-132-157738-s137.pdf]

Supplemental material for

Sphingosine-1-phosphate receptor 1 activation in the central nervous system drives cisplatin-induced cognitive impairment

[1,2]Silvia Squillace, [2,3]Michael L. Niehoff, [1,2]Timothy M. Doyle, [2,4]Michael Green, [5]Emanuela Esposito, [5]Salvatore Cuzzocrea, [1,2,4]Christopher K. Arnatt, [6]Sarah Spiegel, [2,3]Susan A. Farr, and [1,2]Daniela Salvemini

[1]Department of Pharmacology and Physiology and [2]The Henry and Amelia Nasrallah Center for Neuroscience, Saint Louis University School of Medicine, St. Louis, Missouri, USA. [3]Department of Internal Medicine-Geriatrics, Saint Louis School of Medicine, St. Louis, Missouri, USA. [4]Department of Chemistry, Saint Louis University, St. Louis, Missouri, USA. [5]Department of Clinical and Experimental Medicine and Pharmacology, University of Messina, Messina, Italy. [6]Department of Biochemistry and Molecular Biology, Virginia Commonwealth University School of Medicine, and the Massey Cancer Center, Richmond, Virginia, USA.

Address correspondence to: Daniela Salvemini, Department of Pharmacology and Physiology, Saint Louis University School of Medicine, 1402 South Grand Blvd., St. Louis, MO 63104, USA. Phone: 314-977-6430; Email: daniela.salvemini@health.slu.edu

Disclosure of Potential Conflict of Interest: All authors have declared that no conflict of interest exists.

Supplemental Methods

Animals

Seven- to eight-week-old male and female wild-type (WT; C57BL/6) mice were purchase from Envigo. Seven to eight-week old *Tlr4*$^{-/-}$ (B6.B10ScN-Tlr4lps-del/JthJ, strain #007227), *Nlrp3*$^{-/-}$ (B6.129S6-Nlrp3tm1Bhk/J, strain #021302) and WT male mice were purchased from Jackson Laboratory (Bar Harbor, ME, USA). The *Tlr4* knockout mice are crossbred from male and female mice genotyped as homozygous for complete TLR4 deletion (tlr4$^{lps\text{-}del}$) by Jackson Laboratories. The Tlr4$^{Lps\text{-}del}$ spontaneous mutation corresponds to a 74723 base pair deletion that completely removes the Tlr4 coding sequence (for the present work we used the following: breeding req#'s 643318, 669973, 688162, 705278 and 720449). Likewise, the *Nlrp3* knockout mice are crossbred from male and female mice genotyped as homozygous for the replacement of the Nlrp3 coding sequence with a neo cassette by Jackson Laboratories (for the present work we used the following: breeding PenID#'s m1002148, m1082077, m568439, m764440). Experimental validation in the nervous system for both Tlr4 (strain #007227) and Nlrp3 (strain #021302) global knockouts were previously published (1, 2).

Astrocyte-specific *S1pr1* knockout and their wild type littermates were descendants of original homozygous *S1pr1*$^{fl/fl}$*;GFAP-Cre* breeder mice gifted to us by J. Chun (The Scripps Research Institute, La Jolla, CA; (3)) and inbred to maintain their homozygosity for the floxed *S1pr1* gene and hemizygosity for *Gfap-Cre*. The colony was regularly backcrossed with C57BL/6J wild-type mice (Harlan Laboratories) to reduce accrument of inbred genetic defects. The unmodified natural *S1pr1* sequence contains a forward sequence of a non-coding *Edg-1m* region upstream of the *S1pr1* open reading

frame (ORF) and a reverse sequence containing the complimentary sequence for the *S1pr1* ORF flanked by sequences for *B1T1C* and *SPB1*. As described by Choi et al. (3), loxP sites were inserted into positions between Edg-1m (forward) and *SPB1* (reverse) and following the *S1pr1* ORF sequences (forward) before the *B1TIC* site (reverse). In the presence of Cre, the *SPB1* and the entire *S1pr1* ORF sequence are removed (3). The subsequent recombination events place *Edg-1m* (forward) and *B1T1C* (reverse) in proximity to each other (3). All *S1pr1* knockout mice and their wild type littermates were genotyped from DNA samples obtained from ear snips collected after weening and prior to their assignment to treatment groups, as previously described (3, 4). Wild type mice were determined by the presence of a 335 bp product of the *Edg-1m* (forward) and *SPB1* (reverse) sequences, but lacking a 650 bp *Gfap-Cre* PCR product (Supplementary Figure 6). *S1pr1* knockout mice were determined by the presence of a single 335 bp PCR product of the *Edg-1m* (forward) and *SPB1* (reverse) sequences and a 650 bp PCR product for *Gfap-Cre* (Supplementary Figure 6). *S1pr1* deletion was detected by the presence of a 499 bp PCR product for the *Edg-1m* (forward) and *B1T1C* (reverse) recombination (3, 4), indicating that sequences for *SPB1* and the entire *S1pr1* ORF were removed. We have previously validated that presence of the 499 bp deletion product corresponded to at least a 50% reduction in S1PR1 protein expression in the central nervous system in mice with *Gfap-Cre* (4) and Choi et al., demonstrated that in *Gfap-Cre* expressing animals, this construct reduced S1pr1 expression specifically in astrocytes (3). Animals were excluded if DNA from ear snips presented this 499 bp deletion product, indicating that the *Cre* gene had translocated away from the *Gfap* promoter.

All mice were housed 4-6/cage according to their sex in a controlled environment (12-h light-dark cycles) with food and water available *ad libitum*. All experiments were conducted with the experimenters blinded to treatment conditions.

Cisplatin-induced cognitive impairment model

To induce cognitive impairment, cisplatin was administrated as previously described (5). Briefly, mice were given a cycle of daily intraperitoneal (i.p.) injections of cisplatin (2.3 mg/kg, Accord Healthcare, Inc., Durham, NC, USA) or saline for 5 days, rested (no injections) for the following 5 days and then given another 5-day cycle of cisplatin or saline. The cumulative dose of 23 mg/kg of cisplatin used in mice (human equivalent dose 70 mg/m^2) is comparable to the total dose per cycle used in patients (75 mg/m^2). Body weight of mice as well as signs of discomfort, motor impairment or illness were monitored throughout the treatment period.

Tested compounds preparation and administration

FTY720 was purchased from Cayman Chemical (Ann Arbor, MI, USA). Ozanimod was purchased from Med Chem Express (Monmouth Junction, NJ, USA). Mice received i.p. injections of FTY720 (1 mg/kg in saline), ozanimod (1 mg/kg in 10% PEG300 and 90% saline) or their vehicle 15 minutes before each cisplatin injection. The dose of 1mg/kg for the S1PR1 functional antagonists (FTY720 and ozanimod) was identified in our preliminary studies and previous published work as the optimal dose to completely block S1PR1-mediated neuroinflammation and chemotherapy-induced nurotoxicities (4, 6, 7) The dose of 1mg/kg for FTY720 was also shown to suppress breast cancer progression

in mice (8). The TLR4 antagonist, TAK-242, was purchased from Selleck Chemicals LLC (Houston, TX, USA). The NLRP3 antagonist MCC950 was purchased from Cayman Chemical (Ann Arbor, MI, USA). Mice received constant intracerebroventricular infusion of TAK-242 (20µg/day in 10%DMSO, 40% PEG 300, 50% sterile dH2O), MCC950 (5µM in sterile distilled water) or their vehicle starting from 48h before the first cisplatin injection and until the beginning of behavioral tests (that is 2 weeks after the last cisplatin injection). The doses of TAK-242 (preliminary data) and MCC950 (preliminary data and (9)) were previously shown to completely block TLR4 receptor and NLRP3 inflammasome activation, respectively. For our study we used clinically relevant doses that we have calculated based on a guide for dose conversion between mice and humans taking into account the recommended dose for humans (10).

Implant of intracerebroventricular cannula with Alzet micro-osmotic pump

Sterile Alzet micro-osmotic pumps (model 1004, 0.11ul/hr, 28 days) and intracerebroventricular brain infusion kits were prepared and assembled prior to surgical implantation. Mice were anesthetized with 3% isoflurane/100% O2 inhalation, placed in a stereotaxic instrument, and maintained on 2% isoflurane/100% O2 for the duration of surgery. After disinfecting the skin with Nolvasan solution, an incision was made in the midline starting over the central cranial suture and extending down the back of the neck. Using a blunt-tip scissors, a subcutaneous pocket was created extending to the left hindlimb in which to place the Alzet pump. Using the stereotaxic instrument, the cannula is positioned over the bregma and then moved 1.0mm to the right and 0.5mm posterior to the bregma. The cannula driver was then lowered until the 30-gauge stainless steel

tube penetrated the skull and entered the intracerebral ventricle. The cannula was held in place for five minutes to allow the Loctite glue to dry. Once the pump, tubing and cannula are securely in place, the skin was closed using skin clips. Antibiotic ointment was applied to the closed wound, mice removed from the stereotaxic, and placed on a warm absorbent pad until awake. Once fully awake and mobile, mice were individually housed in a clean cage.

Verification of intracerebroventricular infusion

After completion of behavioral assessment, mice were deeply anesthetized (ketamine 110 mg/kg / xylazine 11mg/kg - 0.1 ml/10g of body weight). For the verification of cannula placement, black ink diluted in saline (5μl) was injected through the guide cannula. Mice were then perfused with cold phosphate-buffered saline (PBS), brains removed and cut coronally through the cannula placement. Only the animals with correct cannula placement in the target areas were included in the data analysis.

Behavioral testing

Two weeks after the last dose of cisplatin, mice underwent a test battery of behavioral responses to investigate various aspects of cognitive function. Behavioral assessment was performed between 7:00 and 12:00 hrs. Mice were individually housed 1 week prior to the start of behavioral testing until the end of the study to ensure equal levels of arousal during behavioral testing. Each mouse received only 1 test per day.

T-maze: The T-maze was used to test complex memory task (11) and consisted of a black plastic alley with a start box at one end and two goal boxes at the other. The start

box is separated from the alley by a plastic guillotine door that prevent movement down the alley until raised at the onset of training. An electrifiable floor of stainless-steel rods run throughout the maze to deliver a mild scrambled foot-shock 5 seconds after the guillotine door is raised and a cue buzzer (door-bell type sounded at 55 dB). This test measures retention of the previously learned task of avoiding a mild 0.35 mA foot shock. When measured 1 week after training, the greater the number of trials it took an animal to reach the present criterion of 5 avoidances in 6 consecutive trials, the poorer the long-term memory of the previously learned information regarding the spatial location of the goal box (right/left) and auditory/visual stimulus, indicating long-term hippocampal memory impairment.

Novel object-place recognition (NOPRT): The NOPRT was used to test memory retention of an object it was exposed to 24h prior to testing (12). Mice have an innate preference for novelty, this means they will spend more time exploring a novel object than a familiar one. Each mouse was placed in the test arena (measures) and permitted to explore 2 identical objects for 5 min. After 24h, one of the training objects was replaced with a novel object placed in a different position within the arena. The mouse could explore the 2 objects for another 5 min. The familiar and novel objects were of the same material to avoid potential interference of deficits in sense of touch or smell. Mice that did not explore both objects during the training day were not included in the analysis. The discrimination index was calculated as (time with novel-time with familiar object)/total exploration time of both objects.

Puzzle box: The Puzzle box was used to test complex problem-solving and memory based on the innate preference of mice for small and dark environments. It consists of a

box (61 cm x 30 cm) divided by a barrier into a large bright start chamber and small dark goal box. Access to the goal box is via a tunnel which entrance is completely opened (Trials 1-2), partially blocked by a guillotine (Trials 3-4), blocked by nesting paper (Trials 5-7) or blocked by a T-shaped cardboard plug (Trials 8-11). The test is performed over 4 consecutive days during which mice are challenged with obstacles of increasing difficulty that need to be overcame in a limited amount of time (4 minutes/trial). Mice which take 4 minutes to complete the first 3 trials are excluded from the data analysis.

Open-field test: The Open-field test was used to determine whether cognitive test performance was affected by changes in animal activity due to treatments, mice were allowed to freely roam an empty arena (53 cm x 63 cm) for 5 min and distance traveled in cm was recorded by the ANY-maze (San Diego Instruments, CA, USA).

Elevated Plus Maze test: The elevated plus maze was used to assess potential alterations in the levels of anxiety in our mice. The apparatus consists of 4 arms perpendicular to each other in the shape of a plus sign, elevated 50 cm above the floor. Each arm is 35.5 cm in length; two opposite arms are open while the other two opposite arms are enclosed, as previously described (13). The mouse is placed in the central platform facing an enclosed arm and allowing it to freely explore the maze for 5 min. The time spent in open arms was recorded by the ANY-maze (San Diego Instruments, CA, USA). Anxiety was indicated by decreased time spent in the open arms. The test arena was wiped with a damp cloth after each trial.

Estrus smears

Vaginal smears were taken from female mice treated according to our cisplatin protocol beginning 1 week prior to treatment, throughout treatment regimen and then 10 days after completing cisplatin. Cells were placed on a glass slide and analyzed with a light microscope to determine their stage of estrous cycle. Examination of the smears for cellular morphology and type associated with particular stages of the estrous cycle (14) revealed that while all females cycled normally prior to treatment, 100% of females treated with cisplatin with or without FTY720 or ozanimod experienced a slow-down or pause in estrous cycling during the treatment phase. However, all the females in these groups were freely cycling 10 days after treatment completion.

Sphingolipid measurements by mass spectrometry

As previously described (4, 15), lipids were extracted from hippocampus and PFC tissues, internal standards added, and sphingolipids were quantified by LC-ESI-MS/MS (AB Sciex 5500). Sphingolipid levels were expressed as pmol/mg protein.

Western blot

Cytosolic extract was prepared and separated electrophoretically for transfer to nitrocellulose membranes, as previously described (16). For Western blot of MnSOD and NLRP3, the blots were probed at 4°C overnight in 1× phosphate-buffer saline (Biogenerica srl, Catania, Italy), 5% (w/v) non-fat dried milk, 0.1% Tween-20 and the following primary antibodies: anti-nitrated MnSOD (1:1000; Millipore, code 06984), anti-cleaved Caspase 1 (1:500; Cell Signaling, Danvers, MA, USA, #4199) or anti-NLRP3 (1:500; Abcam, Cambridge, UK, ab263899). Then, membranes were incubated with

peroxidase-conjugated goat anti-mouse IgG secondary antibody (1:2000, Jackson ImmunoResearch, West Grove, PA, USA, #AB_10015289) for 1h at room temperature. Anti-β-actin antibody (1:500; Abcam, Cambridge, UK, ab179467) was used as control. The blots were documented using a ChemiDoc XRS (Bio-Rad, Hercules, CA, USA), and analyzed by using Image Lab 3.0 software (Bio-Rad, Hercules, CA, USA). For Western blots of S1PR1 and TLR4, whole tissue lysates were prepared as previously described (9) and heat denatured for 5 minutes by 95ºC (S1PR1) or 56ºC (TLR4). Protein bands were resolved by SDS-PAGE (4-20%) and transferred to PVDF. Membranes were blocked for 1 h at room temperature in 3% bovine serum albumin in 1X Tris-buffered saline (TBS, pH 7.4) and subsequently probed overnight at 4°C with rabbit anti-S1PR1 (1:20,000; ThermoFisher Scientific, #1040) antibody or rabbit anti-TLR4 (1:1000; Proteintech 19811-1-AP) antibody in 1% bovine serum albumin in 1X TBS (pH 7.4). Blots were washed in TBS (pH 7.4) with 0.1% Tween-20 (TTBS) and bands were visualized by incubation with peroxidase-conjugated goat anti-rabbit IgG (1:10,000, Cell Signaling Technologies, #7074) in TTBS containing 1% bovine serum albumin and enhanced chemiluminescence (Bio-Rad, Hercules CA). Peroxidase were deactivated by treating blots twice with 30% hydrogen peroxide for 15 min (17) and probed with mouse anti-β-actin (1:10,000, Millipore-Sigma, A5441) or mouse anti-α-tubulin (1:5000, Millipore-Sigma, T8203) antibodies. Bands were visualized using peroxidase-conjugated goat anti-mouse IgG (1:1000, SeraCare, #5220-0287) antibodies in TTBS containing 1% bovine serum albumin and enhanced chemiluminescence. Blots were documented and analyzed using a Chemidoc MP documentation system and ImageLab™ software v6.1.0 build 7 (BioRad, Hercules CA).

ELISA Kit Assay for IL-1β and IL-10

The concentrations of IL-1β and IL-10 were measured using commercially available enzyme-linked immunosorbent assay (ELISA) kits (R&D System, Minneapolis, MN) according to the manufacturer's instructions. Briefly, samples were thawed on ice and homogenized in specific lysis buffer; subsequently, the samples were homogenized and centrifuged. Supernatants were collected and stored at -20°C. IL-1β and IL-10 were measured using a microplate reader at 450 nm.

Measurement of SOD activity

MnSOD activity was measured as previously described (18). Briefly, the tissues were homogenized with 10 mM phosphate-buffered saline (pH 7.4) and subsequently centrifuged. To determinate MnSOD activity, a competitive inhibition assay using xanthine oxidase generated O_2 was performed by reducing nitrobluetetrazolium (NTB) to blue tetrazolium salt. The reaction was performed in sodium carbonate buffer (50 mM, pH 10.1) containing EDTA (0.1 mM), nitrobluetetrazolium (25 µM), xanthine (0.1 mM), and xanthine oxidase (2 nM). The rate of NTB reduction was monitored at 560 nm. Enzymatic activity was expressed in units per mg of protein. All determinations were performed in triplicate.

Cisplatin-TLR4 interaction modeling

The TLR4-MD-2 complex structure was obtained from the Protein Data Bank (PDB: 3FXI). All modeling was performed in Schrodinger Maestro 12.9. Once imported, the

TLR4-MD-2 complex was prepared by removing water molecules, lipids, ions, LPS, and the MD-2 subunits from the TLR4 homodimer. Then the TLR4 homodimer was prepared, and energy minimized using Protein Preparation Wizard default parameters and an OPLS3e force field. Cisplatin was then covalently docked into the homodimer protein-protein interface using Glide CovDock by specifying the H431/H456/H458 area for docking. The TLR4 homodimer-cisplatin complex was then minimized using the Prime VSGB2.0 energy model. Figures were generated using Pymol.

Statistical analysis

Data were analyzed using GraphPad Prism version 9.1.0 for Windows (GraphPad Software, San Diego, CA, USA). All values in the figures are expressed as mean ± standard error of the mean (SEM) of N number of animals. Behavioral and biochemical data, except sphingolipidomic data, were analyzed with two-tailed Student's t-test, two-tailed two-way ANOVA with Bonferroni comparisons or two-tailed one-way ANOVA followed by a Dunnett's. A P value less than 0.05 was considered significant for all analyses. Sphingolipidomic data were analyzed by two-tailed Student's *t*-test with the false discovery rate controlled by Benjamini-Hochberg procedure (Q value less than 0.05) or by two-tailed two-way ANOVA with Bonferroni comparisons for each species (significance set at $P<0.05$).

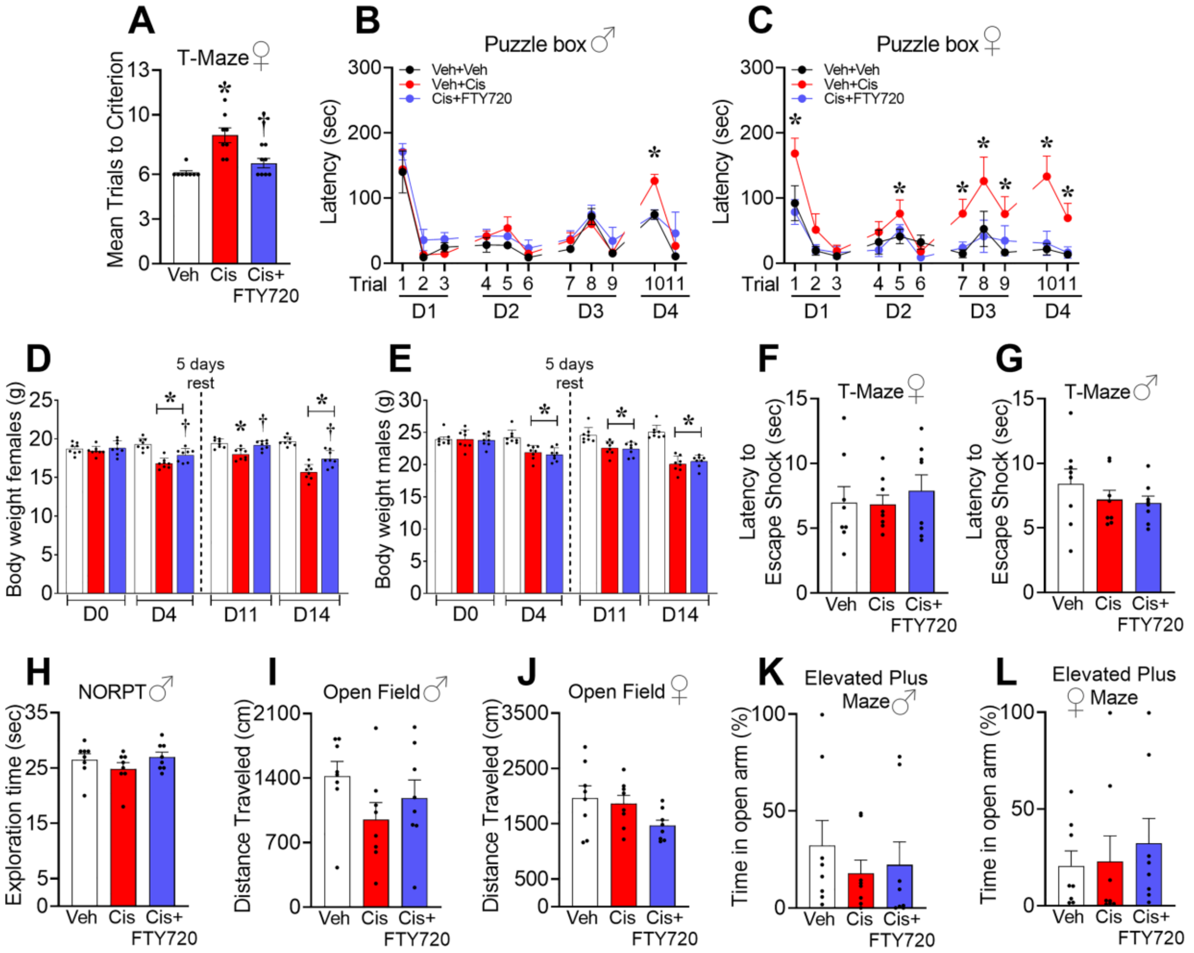

Figure S1. FTY720 attenuated CICI in both males and females with no adverse effect on anxiety-like behavior and locomotor activity. (**A-C**) Female mice receiving cisplatin took more trials to reach criterion in the T-maze (**A**). Both male (**B**) and female (**C**) mice showed problem-solving and memory impairment in the Puzzle box after cisplatin treatment. FTY720 administered in conjunction with cisplatin attenuated cognitive deficits in both male and female mice (A-C). (**D**,**E**) Body weight of female (**D**) and male (**E**) mice was monitored throughout the cisplatin treatment period (I cycle of 5-days injections D0-D4; II cycle of 5-days injections D11-D14). As expected, cisplatin induced a progressive reduction of body weight in both males and females. FTY720 co-treatment partially attenuate body weight loss in females, but not in males. (**F-L**) Cisplatin and FTY720 effects on cognitive functions were not due to reduction in motivation to escape a noxious stimulus (**F** and **G**), overall locomotor activity (**H-J**) or changes in anxiety levels (**K** and **L**). Mean±SEM, n=7-8/group, (**A, F-L**) two-tailed one-way ANOVA with Dunnett's comparisons or (**B-E**) two-tailed two-way ANOVA with Bonferroni's comparisons. *p<0.05 vs. Veh, †p<0.05 vs. Cis.

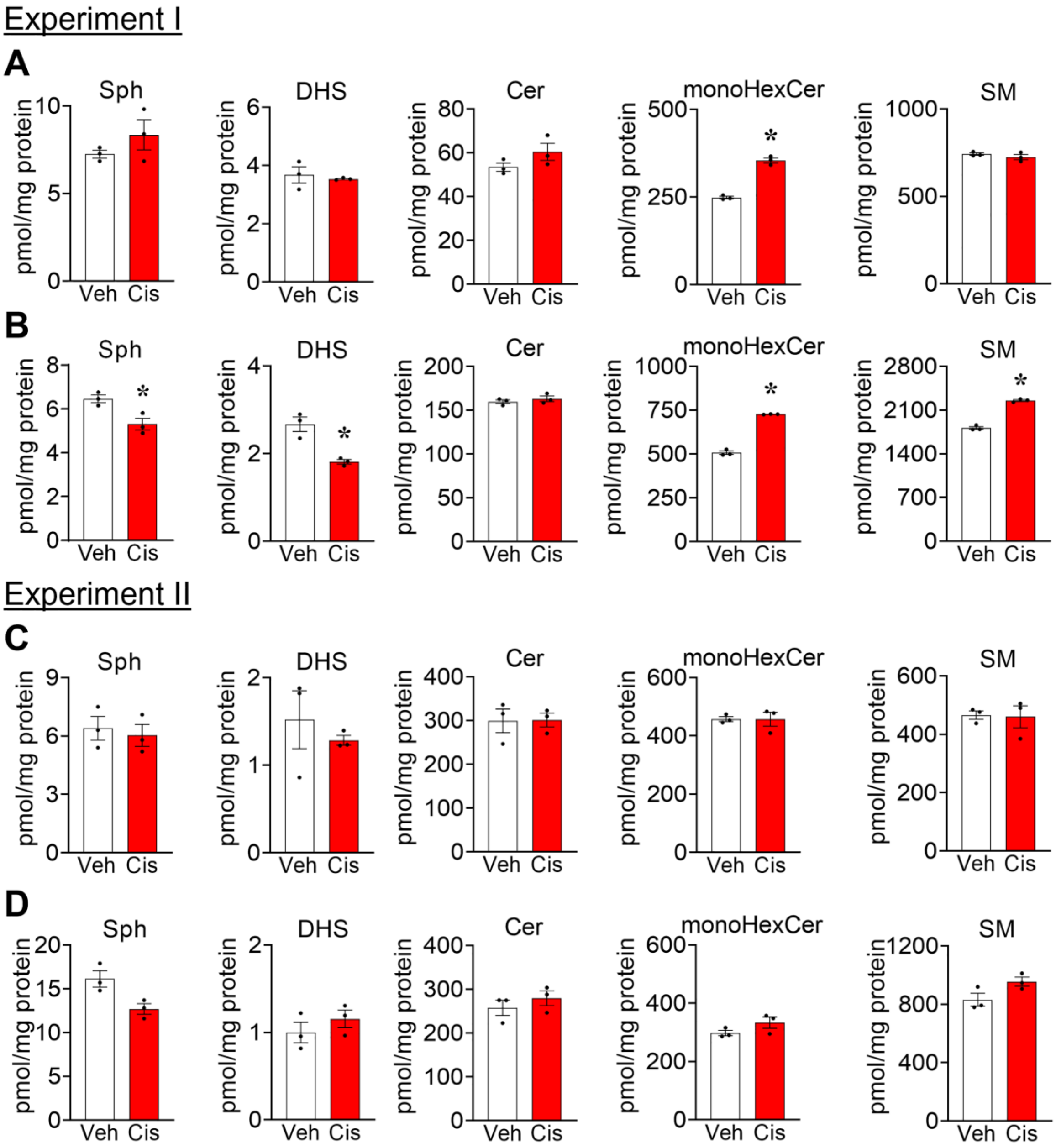

Figure S2. Sphingolipidomic analysis revealed no changes in sphingomyelin nor in the S1P *de novo* biosynthetic pathway. (**A-D**) Lipids were extracted from hippocampus (**A**, experiment I, and **C** experiment II) and prefrontal cortex (**B**, experiment I, and **D** experiment II) of mice treated with vehicle (Veh) or cisplatin (Cis) and sphingosine (Sph), dihydrosphingosine (DHS), ceramides (Cer), monohexosylceramides (MonoHexCer), and sphingomyelins (SM) measured by LC/ESI/MS/MS. Data are total sphingolipid levels. . Mean±SEM, n=3/group/exp, two-tailed unpaired Student's *t*-test followed by Benjamini-Hochberg, *q<0.05 (adjusted p=0.025) vs. Veh

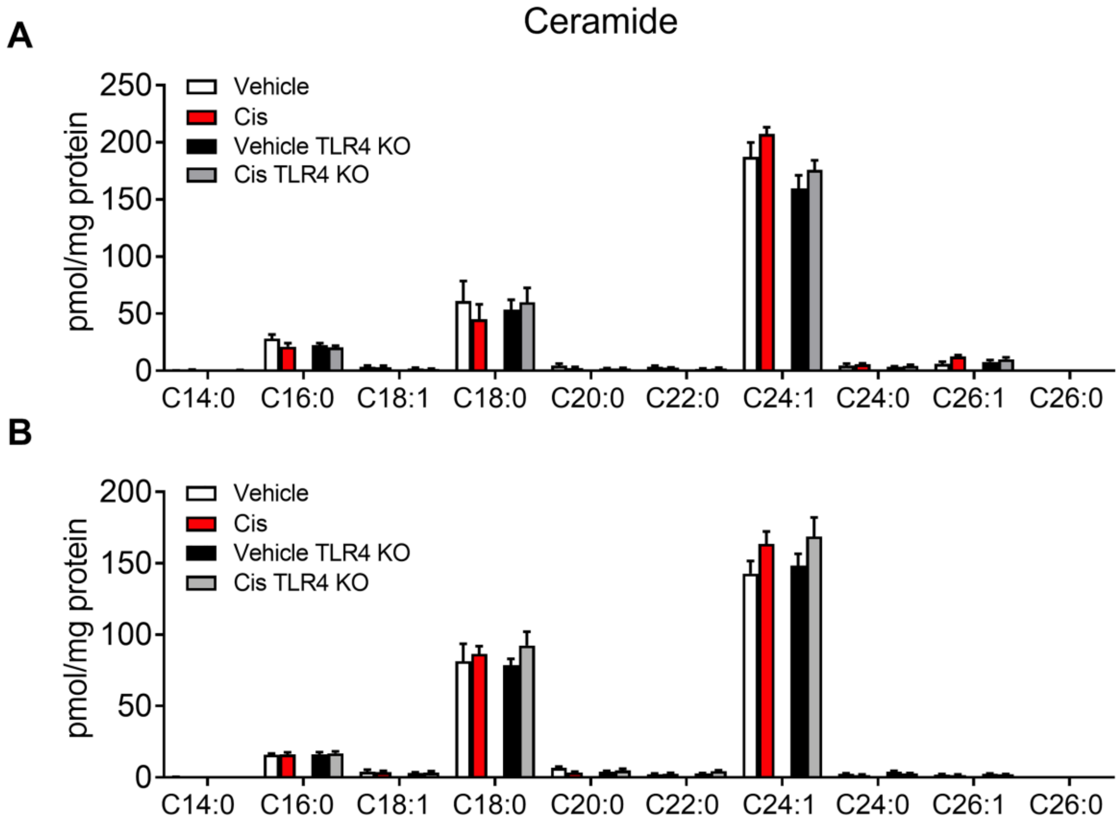

Figure S3. Lack of effects of cisplatin on ceramide levels. Lipids were extracted, from hippocampus (**A**) and prefrontal cortex (**B**) of WT or *Tlr4*$^{-/-}$ mice treated with vehicle (Veh) or cisplatin (Cis) and the indicated ceramide species were determined by LC-ESI-MS/MS. Numbers indicate chain length followed by the number of double bonds in the fatty acid. No significant differences were observed in ceramides between Veh and Cis treated mice. Mean±SEM, n = 3-6/group, two-tailed two-way ANOVA with Bonferroni. No significant differences detected.

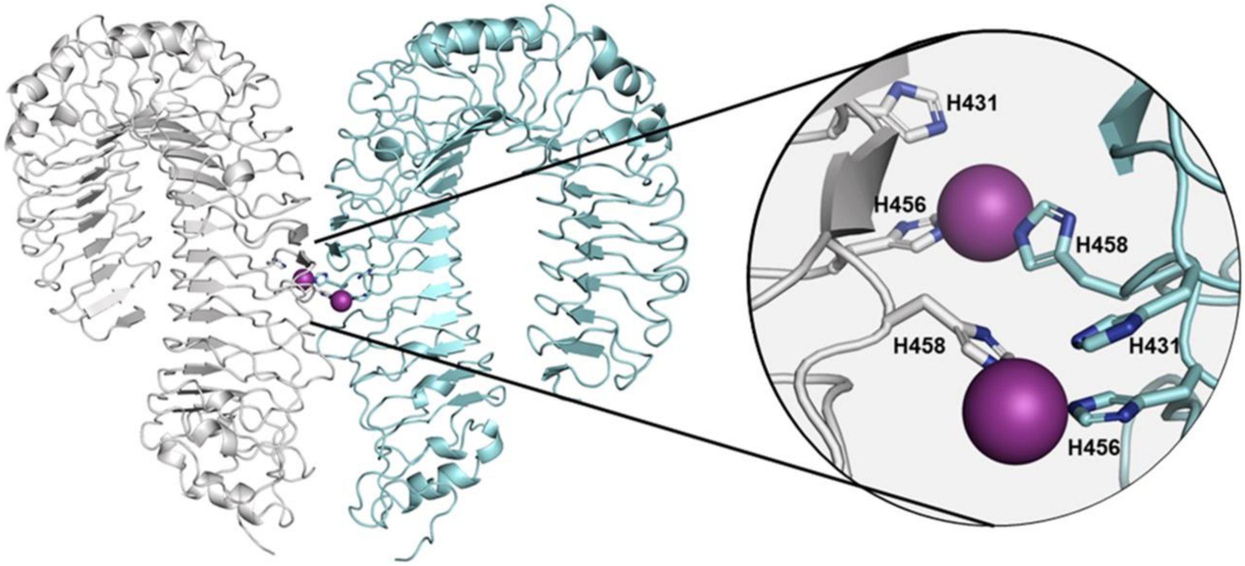

Figure S4. H456 and H458 provide potential binding sites for cisplatin. Homodimer of TLR4 (gray, blue; PDB:3FXI) showing the binding sites at the homodimer interface (MD2 not shown for clarity). The zoomed area shows the model of the two cisplatin (purple) binding sites between opposing H456 and H458 residues. Platination of histidine residues shown to be directed to the Nπ atom of the imidazole rings. The conserved H431 are too far removed to interact with cisplatin or the homodimer interface.

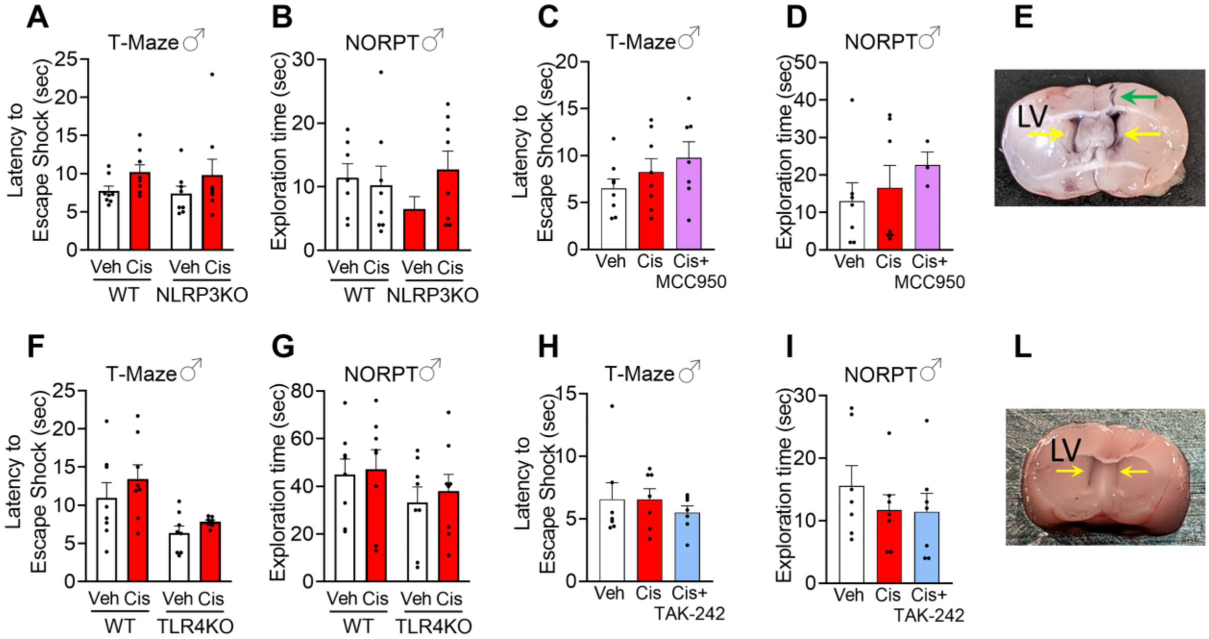

Figure S5. Blocking NLRP3 or TLR4 attenuated CICI without altering overall activity or motivation to escape a noxious stimulus. Cognitive performances of WT, *Nlrp3*$^{-/-}$ (NLRP3KO) and *Tlr4*$^{-/-}$ (TLR4KO) mice receiving either vehicle (Veh) or cisplatin (Cis) were not due to reduction of motivation to escape a noxious stimulus in the T-maze (**A** and **F**) or impairments in locomotor activity during NORPT (**B** and **G**) Mean±SEM, n=7-8/group. In WT mice, MCC950 and TAK-242 beneficial effects on cognitive functions following cisplatin did not alter mice capability to escape a noxious stimulus during T-Maze (**C** and **H**) or overall activity during NORPT (**D** and **I**) Mean±SEM, n=3-7/group. Correct placement of intracerebroventricular cannulas has been verified after completion of MCC950 (**E**) and TAK-242 (**L**) experiments. LV= lateral ventricle; green arrow indicates black ink filling the injection site; yellow arrows indicate black ink filling lateral ventricles. Data analyzed by two-tailed one-way ANOVA with Dunnett's comparisons, *P<0.05.

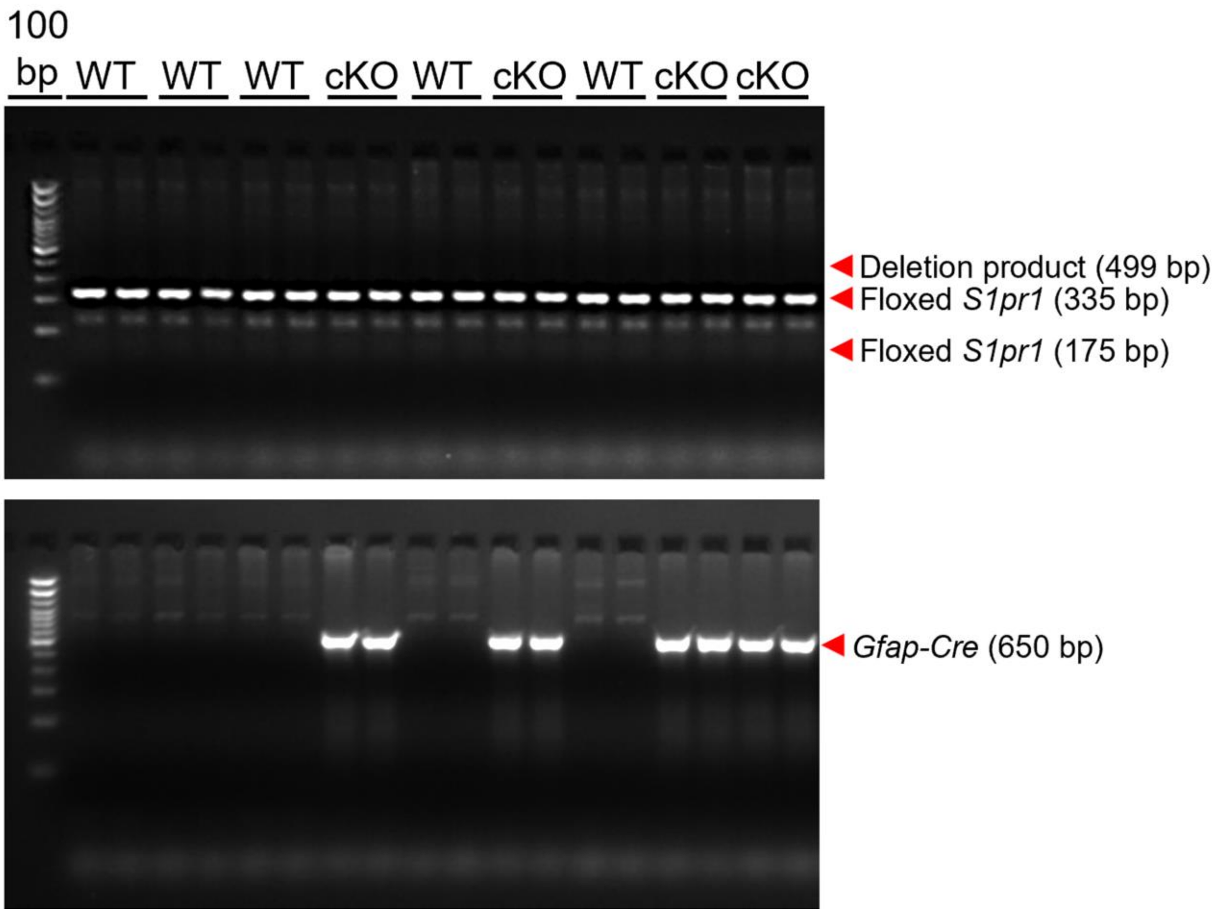

Figure S6. DNA agarose gel electrophoresis of PCR for genotyping astrocyte-specific *S1pr1* knockout and control mice. DNA was isolated from ear snips after weening and subjected to PCR for floxed *S1pr1* allele (*S1pr1*$^{fl/fl}$, 335 bp), wild type allele (175 bp), deletion product (499 bp) and *Gfap-Cre* (650 bp). Image is an example genotyping assay of a single litter of male and female mice used in behavior experiments. WT = wild type mouse expressing S1pr1 (*S1pr1*$^{fl/fl}$) , cKO = astrocyte-specific conditional knockout mouse (*S1pr1*$^{fl/fll}$;*Gfap-Cre*), 100 bp = 100 base pair ladder.

1. He X-f, Li L-l, Xian W-b, Li M-y, Zhang L-y, Xu J-h, et al. Chronic colitis exacerbates NLRP3-dependent neuroinflammation and cognitive impairment in middle-aged brain. *Journal of Neuroinflammation.* 2021;18(1):153.
2. Wu Y, Wang Y, Wang J, Fan Q, Zhu J, Yang L, et al. TLR4 mediates upregulation and sensitization of TRPV1 in primary afferent neurons in 2,4,6-trinitrobenzene sulfate-induced colitis. *Molecular pain.* 2019;15:1744806919830018.
3. Choi JW, Gardell SE, Herr DR, Rivera R, Lee CW, Noguchi K, et al. FTY720 (fingolimod) efficacy in an animal model of multiple sclerosis requires astrocyte sphingosine 1-phosphate receptor 1 (S1P1) modulation. *Proceedings of the National Academy of Sciences of the United States of America.* 2011;108(2):751-6.
4. Stockstill K, Doyle TM, Yan X, Chen Z, Janes K, Little JW, et al. Dysregulation of sphingolipid metabolism contributes to bortezomib-induced neuropathic pain. *J Exp Med.* 2018;215(5):1301-13.
5. Zhou W, Kavelaars A, and Heijnen CJ. Metformin Prevents Cisplatin-Induced Cognitive Impairment and Brain Damage in Mice. *PloS one.* 2016;11(3):e0151890.
6. Chen Z, Doyle TM, Luongo L, Largent-Milnes TM, Giancotti LA, Kolar G, et al. Sphingosine-1-phosphate receptor 1 activation in astrocytes contributes to neuropathic pain. *Proceedings of the National Academy of Sciences of the United States of America.* 2019;116(21):10557-62.
7. Janes K, Little JW, Li C, Bryant L, Chen C, Chen Z, et al. The development and maintenance of paclitaxel-induced neuropathic pain require activation of the sphingosine 1-phosphate receptor subtype 1. *The Journal of biological chemistry.* 2014;289(30):21082-97.
8. Nagahashi M, Yamada A, Katsuta E, Aoyagi T, Huang WC, Terracina KP, et al. Targeting the SphK1/S1P/S1PR1 Axis That Links Obesity, Chronic Inflammation, and Breast Cancer Metastasis. *Cancer research.* 2018;78(7):1713-25.
9. Doyle TM, Chen Z, Durante M, and Salvemini D. Activation of Sphingosine-1-Phosphate Receptor 1 in the Spinal Cord Produces Mechanohypersensitivity Through the Activation of Inflammasome and IL-1β Pathway. *J Pain.* 2019;20(8):956-64.
10. Nair AB, and Jacob S. A simple practice guide for dose conversion between animals and human. *J Basic Clin Pharm.* 2016;7(2):27-31.
11. Farr SA, Banks WA, La Scola ME, Flood JF, and Morley JE. Permanent and temporary inactivation of the hippocampus impairs T-maze footshock avoidance acquisition and retention. *Brain Res.* 2000;872(1-2):242-9.
12. Hammond RS, Tull LE, and Stackman RW. On the delay-dependent involvement of the hippocampus in object recognition memory. *Neurobiol Learn Mem.* 2004;82(1):26-34.
13. Farr SA, Niehoff ML, Kumar VB, Roby DA, and Morley JE. Inhibition of Glycogen Synthase Kinase 3β as a Treatment for the Prevention of Cognitive Deficits after a Traumatic Brain Injury. *J Neurotrauma.* 2019;36(11):1869-75.
14. Byers SL, Wiles MV, Dunn SL, and Taft RA. Mouse estrous cycle identification tool and images. *PloS one.* 2012;7(4):e35538.
15. Hait NC, Allegood J, Maceyka M, Strub GM, Harikumar KB, Singh SK, et al. Regulation of histone acetylation in the nucleus by sphingosine-1-phosphate. *Science (New York, NY).* 2009;325(5945):1254-7.
16. Casili G, Ardizzone A, Basilotta R, Lanza M, Filippone A, Paterniti I, et al. The Protective Role of Prolyl Oligopeptidase (POP) Inhibition in Kidney Injury Induced by Renal Ischemia-Reperfusion. *Int J Mol Sci.* 2021;22(21).

17. Sennepin AD, Charpentier S, Normand T, Sarré C, Legrand A, and Mollet LM. Multiple reprobing of Western blots after inactivation of peroxidase activity by its substrate, hydrogen peroxide. *Anal Biochem.* 2009;393(1):129-31.
18. Ilari S, Dagostino C, Malafoglia V, Lauro F, Giancotti LA, Spila A, et al. Protective Effect of Antioxidants in Nitric Oxide/COX-2 Interaction during Inflammatory Pain: The Role of Nitration. *Antioxidants (Basel, Switzerland).* 2020;9(12).
